# Gender differences in the association between the triglyceride-glucose index and peripheral artery disease in vascular surgery inpatients aged 50 and above: a retrospective cross-sectional study

**DOI:** 10.3389/fendo.2025.1578025

**Published:** 2025-08-20

**Authors:** Zhi-wei Li, Peng Zhou, Bei-hao Shi, Ya-qin Gong, Ke Lu, Jian Zhu

**Affiliations:** ^1^ Department of Vascular Surgery, Gusu School, Nanjing Medical University, The First People’s Hospital of Kunshan, Suzhou, Jiangsu, China; ^2^ Department of Vascular Surgery, Affiliated Kunshan Hospital of Jiangsu University, Suzhou, Jiangsu, China

**Keywords:** TyG index, peripheral artery disease, gender differences, insulin resistance, atherosclerosis

## Abstract

**Background:**

Peripheral artery disease (PAD) is a critical manifestation of systemic atherosclerosis, significantly affecting vascular health. Insulin resistance (IR) plays a central role in PAD pathophysiology. The triglyceride-glucose index (TyGI), a recognized marker of IR, has limited evidence regarding its association with PAD. This study aimed to investigate the relationship between the TyGI and PAD among vascular surgery inpatients aged over 50 in Kunshan, China, offering insights into clinical prevention and risk assessment of PAD.

**Methods:**

A retrospective cross-sectional study was conducted on 5923 patients (aged ≥ 50 years) admitted to the vascular surgery department of the Affiliated Kunshan Hospital of Jiangsu University, Suzhou, China, from December 2017 to August 2024. The TyGI was the exposure variable, while PAD, defined as PAD-like symptoms with an ankle-brachial index (ABI) < 0.9, was the outcome variable. Covariates included age, sex, body mass index (BMI), smoking status, alcohol consumption, hypertension, diabetes, low-density lipoprotein (LDL), total cholesterol (TC), alanine aminotransferase (ALT), and neutrophil counts (NEUT). Multiple logistic regression, subgroup analysis, curve fitting, and threshold effect analysis were performed.

**Results:**

After adjusting for covariates, the TyGI showed a significant positive association with PAD (OR = 1.92, 95% CI: 1.50–2.45, *P* < 0.001). When classified by TyGI quartiles, individuals in the highest quartile had a significantly increased risk of PAD (OR = 2.19, 95% CI: 1.43–3.35, *P* < 0.001). Subgroup analysis revealed a significant interaction effect of gender on the TyGI-PAD relationship (*P* for interaction < 0.05). In male patients, the TyGI showed a positive linear correlation with PAD, whereas in female patients, a positive nonlinear relationship was observed, with a threshold value of 9.68.

**Conclusion:**

This study demonstrates a significant positive association between TyGI and PAD in vascular surgery inpatients aged 50 and older, with a gender-specific difference in the nature of this relationship. A linear correlation was found in male patients, while in female patients, the association was nonlinear, with a threshold effect. These findings provide relevant evidence for understanding the role of the TyGI in peripheral vascular health and could aid in the clinical assessment and management of PAD.

## 1 Introduction

Peripheral artery disease (PAD) is a significant clinical manifestation of systemic atherosclerosis and is strongly associated with increased cardiovascular morbidity and mortality. The clinical presentation of PAD varies, ranging from asymptomatic disease to intermittent claudication and severe limb ischemia, which may ultimately require amputation if untreated (1, 2). With its significantly high prevalence (3), PAD poses a considerable burden on public health, leading to significant disability and reduced quality of life. Despite advances in diagnostic techniques and therapeutic options, the pathophysiological mechanisms underlying PAD remain incompletely understood. This gap in understanding emphasizes the importance of identifying reliable biomarkers for early diagnosis, risk stratification, and timely intervention to slow atherosclerosis progression (4).

PAD is a multifactorial condition influenced by numerous traditional cardiovascular risk factors, including hypertension, diabetes, dyslipidemia, and smoking (5, 6). Among these, insulin resistance (IR) has been recognized as a key factor in PAD pathogenesis. IR, characterized by impaired insulin sensitivity in peripheral tissues (7), is a hallmark of metabolic syndrome and type 2 diabetes, both of which are strongly associated with atherosclerosis and its complications (8, 9). In recent years, the triglyceride-glucose index (TyGI), calculated from fasting triglyceride and glucose levels, has emerged as a reliable marker for IR (10, 11). The TyG index, by incorporating the product of triglyceride (TG) and fasting blood glucose (FBG), provides a more comprehensive reflection of insulin sensitivity. Published studies have demonstrated that the TyG index shows a significant negative correlation with the total glucose disposal rate (M) measured by euglycemic-hyperinsulinemic clamp (r = -0.681), with consistent performance across subgroups stratified by sex, obesity status, and diabetic conditions (12). Furthermore, some studies indicate that the TyG index exhibits greater diagnostic efficacy for metabolic syndrome compared to FBG and TG alone (13). Additionally, the TyG index has been shown to outperform individual measures such as FBG or TG in predicting atherosclerotic cardiovascular disease (ASCVD) events and all-cause mortality (14), likely due to their association with systemic atherosclerosis (15). Importantly, it is simple to implement in clinical practice and cost-effective. Therefore, the TyG index serves as a valuable tool for the prediction and prevention of PAD.

Although several studies have examined the association between TyGI and PAD in different populations, including individuals with type 2 diabetes and the general U.S. adults (16–20), evidence regarding this relationship in Chinese patients undergoing vascular surgery is limited. Furthermore, PAD risk factors and disease progression may vary across populations due to genetic, environmental, and lifestyle differences. Therefore, the primary objective of this study is to investigate the potential association between TyGI and PAD in vascular surgery inpatients aged 50 years and older in China, to provide insights for early clinical assessment and management of PAD.

## 2 Materials and methods

### 2.1 Ethical consideration

This study was approved by the Ethical Board of the Affiliated Kunshan Hospital of Jiangsu University in Suzhou, China (approval # 2025-03-001-H00-KOl) and adhered to the principles outlined in the Declaration of Helsinki. The data used in this study were provided by the hospital and originally collected for clinical purposes. At the time of collection, informed consent was obtained from each participant, with explicit permission for data use in scientific research. The research team received authorization to access and utilize the data in 2025. Throughout the study, we strictly adhered to privacy protection protocols, and all data were anonymized.

### 2.2 Research participants and design

**Figure 1:**
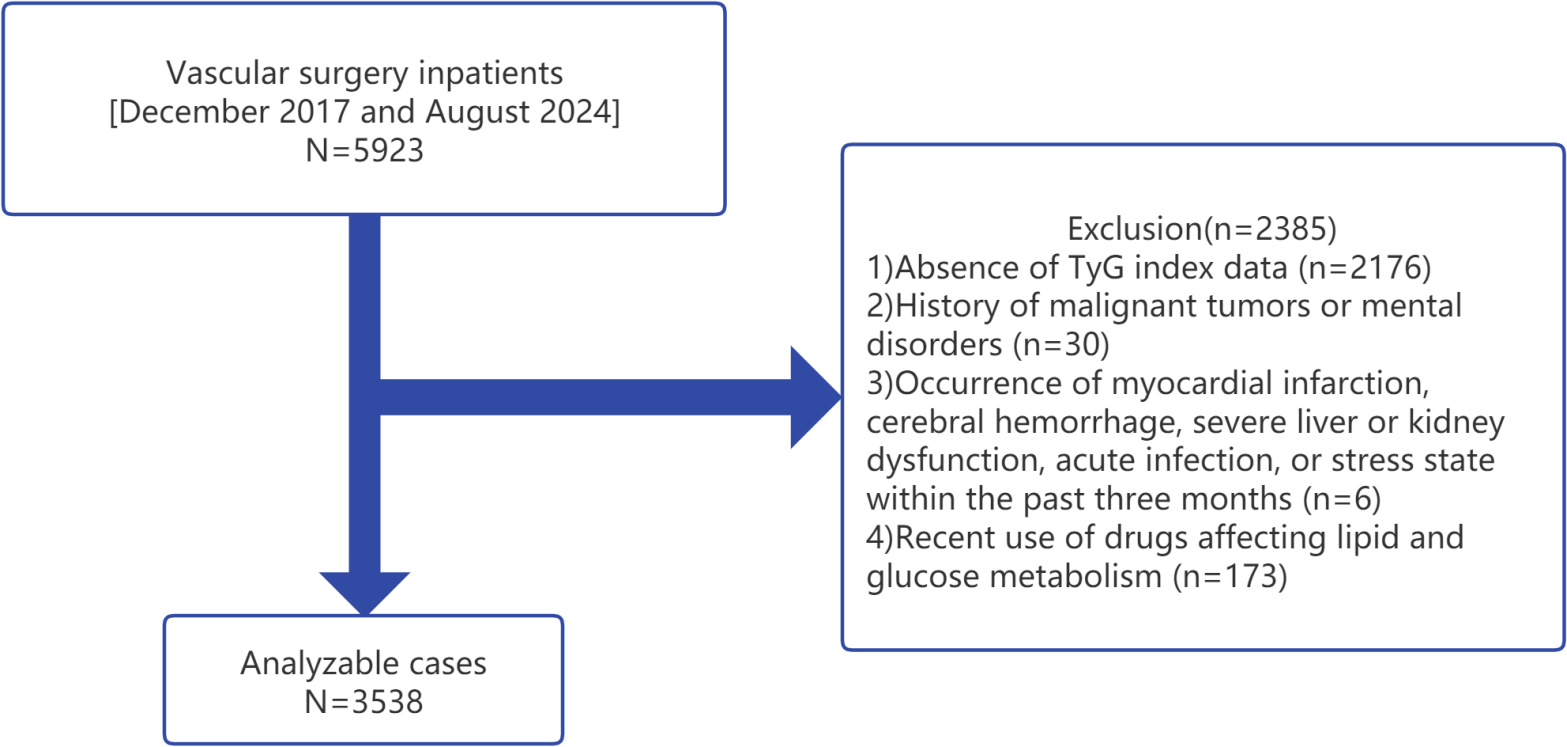
A diagrammatic representation of the study’s design. TyG, triglyceride-glucose index.

A retrospective cross-sectional analysis was conducted on data collected from vascular surgery inpatients aged 50 years and older at the Affiliated Kunshan Hospital of Jiangsu University, Suzhou, China, between December 2017 and August 2024. A total of 5923 patients were initially considered for the study. Medical records were retrieved from the hospital’s database, and all participants had undergone routine blood tests during their hospitalization. The inclusion criteria were: 1) inpatients aged 50 years or older; and 2) availability of complete clinical and biochemical data, including FBG and TG levels. The exclusion criteria were as follows: 1) absence of TyGI data; 2) history of malignant tumors or mental disorders; 3) recent history (within the past three months) of myocardial infarction, cerebral hemorrhage, severe liver or kidney dysfunction, acute infection, or a stress state; and 4) recent use of medications affecting lipid and glucose metabolism (17). After applying the exclusion criteria, 2385 patients were excluded, resulting in a final sample size of 3538 patients for analysis (**Figure 1**).

### 2.3 Exposure and outcome variables

The exposure variable in this study was the TyGI, a reliable marker of IR. The TyGI was calculated using the formula:


$$\text{TyGI}=\text{ln}\left[\frac{\text{TG }\left(\text{mg}/\text{dL}\right)\times \text{FBG }\left(\text{mg}/\text{dL}\right)}{2}\right]$$

FBG was measured using the hexokinase method, while TG was measured using the glycerol phosphate oxidase-phenol aminophenazone (GPO-POD) method. All biochemical measurements were performed using a Beckman AU5800 automated biochemistry analyzer.

The outcome variable was PAD, which was diagnosed based on symptom assessment and ankle-brachial index (ABI) measurement. Patients were initially assessed for PAD-like symptoms according to criteria established by the Task Force of the Society for Vascular Surgery and the International Society for Cardiovascular Surgery (21, 22). ABI measurements were conducted by experienced clinicians using standardized procedures. PAD was defined as the presence of PAD-like symptoms with an ABI < 0.9 (23). All clinical and biochemical parameters were evaluated in the fasting state within three days of admission to ensure data accuracy and consistency.

### 2.4 Covariate variables

The measured covariates included age, sex, body mass index (BMI), total cholesterol (TC), low-density lipoprotein (LDL), alanine aminotransferase (ALT), neutrophil count (NEUT), hypertension, diabetes, smoking status, and alcohol consumption. BMI was calculated as weight (kg) divided by the square of height (m²). TC was measured using an automated enzymatic method, LDL levels were quantified using the Reagent For Low Density Lipoprotein Cholesterol Test Kit (KINGSBIO, Haier Gene) based on a homogeneous enzymatic assay, which involves surfactant-mediated solubilization of HDL-C, VLDL-C, and chylomicrons to release cholesterol for enzymatic detection, and ALT levels were quantified using enzymatic assays. NEUT was assessed using flow cytometry with nuclear staining on the Sysmex XN-10 (B4) hematology analyzer. Hypertension and diabetes were defined as cases previously diagnosed by a medical institution. Smoking was defined as smoking at least one cigarette daily in the past 30 days (24). Drinking was defined as consuming alcohol at least once per week in the past 6 months (25). All clinical and biochemical parameters were evaluated in the fasting state within three days of patient admission to ensure consistency across measurements.

### 2.5 Statistics

Categorical variables were presented as frequencies (percentages), while continuous variables were expressed as means ± standard deviation (SD) for normally distributed data or medians with interquartile ranges (IQRs) for non-normally distributed data. Univariate analyses were performed using Pearson’s chi-squared test or Fisher’s exact test for categorical variables. For continuous variables, independent samples *t*-tests were applied for normally distributed data, while the Mann-Whitney *U* test was used for non-normally distributed data.

Univariate analysis was initially conducted to identify potential associations between PAD and patient characteristics. Partial missing data were observed for the variables “smoking” and “alcohol consumption.” A separate group was created for patients with missing data to address these, ensuring that the adjusted corresponding variable was incorporated into the statistical models without affecting the overall accuracy and reliability of the analysis.

To explore the independent association between TyGI levels and PAD prevalence, generalized estimating equations (GEE) were applied. Three statistical models were developed: Model 1 (unadjusted), Model 2 (minimally adjusted for key covariates), and Model 3 (fully adjusted for all covariates). Variance inflation factor (VIF) analysis was conducted to assess potential collinearity among covariates. Covariates were included in the final model if they met either of the following criteria: 1) the odds ratio (OR) changed by ≥10% when the covariate was added or removed from the model, and 2) the *P*-value for the covariate in the univariate or Model 1 was ≤ 0.1 (26).

In this study, the missing TyGI data was primarily due to manual data loss. To evaluate the potential impact of missing data on our findings, we provided the characteristics of the excluded patients and compares their key features with those of the included cohort, thus assessing possible bias. Additionally, we performed sensitivity analyses using random forest multiple imputation.

Subgroup analyses were performed by stratifying patients according to specific covariates. Interaction effects and subgroup variations were evaluated using the likelihood ratio test (LRT). A generalized additive model (GAM) was used to assess potential nonlinear relationships. If nonlinear associations were observed, piecewise logistic regression models were employed to estimate threshold effects. The inflection points for nonlinear curves were determined using a recursive maximum likelihood estimation approach.

We performed *a priori* power analysis using GPower to assess sample size adequacy, with standard parameters (α = 0.05, power = 0.80). For the primary analysis, targeting an odds ratio (OR) of 1.92, the calculated required sample size was 1,489, which was substantially smaller than our actual sample size, ensuring adequate statistical power. Similarly, for the female subgroup analysis (target OR = 1.37), the minimum required sample size was 984, which we also exceeded. However, in certain smaller subgroups, the sample sizes were insufficient to meet the *a priori* power requirements, potentially affecting the robustness of the subgroup analyses.

All statistical analyses were conducted using the Empower Stats package (X&Y Solutions, Inc., MA, USA) and R software. A two-sided *P*-value of less than 0.05 was considered statistically significant.

## 3 Results

### 3.1 Clinical and demographic traits of subjects

Between December 2017 and August 2024, a total of 3,538 hospitalized vascular surgery patients aged 50 and older were included in the study. The baseline characteristics of these participants, stratified by TyGI quartiles (Q1–Q4), are presented in **Table 1**. The mean age of the patients was 66.29 ± 9.13 years, with a male-to-female ratio of 55.77% to 44.23%. Among the participants, the prevalence of PAD was 8.90%, while the mean TyGI across all participants was 8.62 ± 0.60. Significant trends were observed across TyGI quartiles for key clinical and biochemical parameters, including BMI, TC, TG, FBG, LDL, ALT, and NEUT (*P* < 0.05). Patients in the higher TyGI quartiles had an increased prevalence of hypertension and diabetes (*P* < 0.05). These findings suggest that elevated TyGI levels may be associated with higher metabolic risk profiles and comorbidities.

**Table 1:** Patient characteristics based on TyG index quartiles.

Mean±SD / N (%)						
TyG index	Q1	Q2	Q3	Q4	P-value	P-value*
N	885	884	884	885		
TyG index	7.90±0.23	8.39±0.11	8.77±0.11	9.41±0.40	<0.001	<0.001
Age, years	67.63±9.85	66.84±9.05	65.55±8.51	65.14±8.87	<0.001	<0.001
BMI, kg/m²	23.02±3.24	24.00±3.08	24.83±3.37	25.33±3.10	<0.001	<0.001
TC, mmol/L	4.16±0.93	4.43±0.97	4.69±1.05	4.90±1.04	<0.001	<0.001
TG, mmol/L	0.68±0.15	1.03±0.19	1.44±0.28	2.55±1.33	<0.001	<0.001
LDL, mmol/L	2.32±0.68	2.60±0.74	2.89±0.83	3.09±0.81	<0.001	<0.001
ALT, U/L	21.47±15.78	22.85±18.19	22.86±14.57	26.20±20.26	<0.001	<0.001
N (%)						
Sex, N (%)					<0.001	-
Female	326 (36.84%)	377 (42.65%)	440 (49.77%)	422 (47.68%)		
Male	559 (63.16%)	507 (57.35%)	444 (50.23%)	463 (52.32%)		
Smoking, N (%)					0.084	-
No	583 (65.88%)	612 (69.23%)	603 (68.21%)	620 (70.06%)		
Yes	77 (8.70%)	81 (9.16%)	64 (7.24%)	85 (9.60%)		
Unknown	225 (25.42%)	191 (21.61%)	217 (24.55%)	180 (20.34%)		
Drinking, N (%)					0.059	-
No	612 (69.15%)	646 (73.08%)	629 (71.15%)	647 (73.11%)		
Yes	48 (5.42%)	47 (5.32%)	38 (4.30%)	58 (6.55%)		
Unknown	225 (25.42%)	191 (21.61%)	217 (24.55%)	180 (20.34%)		
Hypertension, N (%)					<0.001	-
No	598 (67.57%)	558 (63.12%)	558 (63.12%)	507 (57.29%)		
Yes	287 (32.43%)	326 (36.88%)	326 (36.88%)	378 (42.71%)		
Diabetes, N (%)					<0.001	-
No	848 (95.82%)	818 (92.53%)	799 (90.38%)	709 (80.11%)		
Yes	37 (4.18%)	66 (7.47%)	85 (9.62%)	176 (19.89%)		
PAD, %					0.486	-
No	808(91.30%)	809(91.52%)	811(91.74%)	795(89.83%)		
Yes	77(8.70%)	75(8.48%)	73(8.26%)	90(10.17%)		

TyG, triglyceride-glucose index; SD, standard deviation; Q1, first quartile; Q2, second quartile; Q3, third quartile; Q4, fourth quartile; BMI, body mass index; TC, total cholesterol; TG, triglyceride; LDL, low density lipoprotein; ALT, alanine aminotransferase; NEUT, medium fine granulocyte count; FBG, fasting blood glucose; PAD, peripheral artery disease.

P-value*: Kruskal Wallis Rank Test for continuous variables, Fisher Exact for categorical variables with Expects<10.

### 3.2 Univariate analysis of factors linked with PAD

The univariate analysis examined the associations between various demographic, clinical, and biochemical variables with PAD. Significant factors associated with PAD included age, sex, BMI, TC, LDL, NEUT, FBG, TyGI, hypertension, diabetes, and smoking (**Table 2**). No significant correlations were observed for ALT, alcohol consumption, or the TG in univariate analysis. These findings indicate that several traditional cardiovascular risk factors are significantly associated with PAD.

**Table 2:** Univariate analysis for PAD.

Characteristics	Statistics	OR (95% CI) P-value
Age, years	66.29±9.13	1.11 (1.10, 1.13) <0.001
BMI, kg/m²	24.32±3.31	0.81 (0.78, 0.85) <0.001
TC, mmol/L	4.55±1.04	0.51 (0.45, 0.58) <0.001
TG, mmol/L	1.43±0.99	1.01 (0.90, 1.13) 0.855
LDL, mmol/L	2.72±0.82	0.46 (0.39, 0.54) <0.001
ALT, U/L	23.35±17.42	0.99 (0.99, 1.00) 0.144
NEUT, 10^9/L	3.75±1.71	1.23 (1.17, 1.30) <0.001
FBG, mmol/L	5.82±1.63	1.23 (1.17, 1.29) <0.001
TyG index	8.62±0.60	1.25 (1.04, 1.50) 0.020
Sex, N (%)		
Female	1565 (44.23%)	Reference
Male	1973 (55.77%)	2.51 (1.93, 3.26) <0.001
Smoking, N (%)		
No	2418 (68.34%)	Reference
Yes	307 (8.68%)	2.27 (1.62, 3.18) <0.001
Unknown	813 (22.98%)	1.17 (0.88, 1.55) 0.280
Drinking, N (%)		
No	2534 (71.62%)	Reference
Yes	191 (5.40%)	0.74 (0.41, 1.32) 0.305
Unknown	813 (22.98%)	1.01 (0.77, 1.33) 0.928
Hypertension, N (%)		
No	2221 (62.78%)	Reference
Yes	1317 (37.22%)	4.49 (3.49, 5.77) <0.001
Diabetes, N (%)		
No	3174 (89.71%)	Reference
Yes	364 (10.29%)	5.94 (4.55, 7.74) <0.001
BMI, kg/m², N (%)		
≤ 24.0	1630 (46.07%)	Reference
>24.0, ≤ 28.0	1437 (40.62%)	0.43 (0.32, 0.58) <0.001
>28.0	471 (13.31%)	0.23 (0.13, 0.42) <0.001

PAD, peripheral artery disease; BMI, body mass index; TC, total cholesterol; TG, triglyceride; LDL, low density lipoprotein; ALT, alanine aminotransferase; NEUT, medium fine granulocyte count; FBG, fasting blood glucose; TyG, triglyceride-glucose index.

### 3.3 Exploration of the connection between the TyGI and PAD

The association between the TyGI and PAD was further explored using multivariate logistic regression models. The results of these analyses are summarized in **Table 3**, with three models applied to evaluate the robustness of the findings: Model 1, Model 2, and Model 3 (**Table 3**). In Model 3, which fully adjusted for potential confounders, the TyGI was significantly and positively associated with PAD (OR = 1.92, 95% CI: 1.50–2.45, *P* < 0.001). When stratified by TyGI quartiles and using the first quartile as a reference, no statistically significant association was found in Model 1. However, in Models 2 and 3, participants in the highest TyGI quartile demonstrated the highest risk of PAD (*P* < 0.05 in both models). In the final model, the OR (95% CI) for PAD in the second, third, and fourth TyGI quartiles, compared with the first quartile, were 1.33 (95% CI: 0.90–1.96, *P* = 0.151), 1.85 (95% CI: 1.23–2.78, *P* = 0.003), and 2.19 (95% CI: 1.43–3.35, *P* < 0.001), respectively.

**Table 3:** Association between TyG index and PAD in different models.

TyG index	Cases/n	Model 1^a^	Model 2^b^	Model 3^c^
		OR (95%CI) P-value	OR (95%CI) P-value	OR (95%CI) P-value
Per 1 SD (0.60)	315/3538	1.25 (1.03, 1.51) 0.020	1.75 (1.44, 2.13) <0.001	1.92 (1.50, 2.45) <0.001
Q1	77/885	1.0	1.0	1.0
Q2	75/884	0.97 (0.70, 1.36) 0.871	1.17 (0.83, 1.67) 0.374	1.33 (0.90, 1.96) 0.151
Q3	73/884	0.95 (0.68, 1.32) 0.738	1.51 (1.05, 2.16) 0.026	1.85 (1.23, 2.78) 0.003
Q4	90/885	1.19 (0.86, 1.64) 0.291	1.95 (1.38, 2.75) <0.001	2.19 (1.43, 3.35) <0.001

a No adjustment.

b Adjusted for age, sex.

c Adjusted for age, sex, BMI, TC, LDL, ALT, NEUT, smoking, drinking, hypertension, diabetes.

TyG, triglyceride-glucose; PAD, peripheral artery disease; BMI, body mass index; TC, total cholesterol; LDL, low density lipoprotein; ALT, alanine aminotransferase; NEUT, medium fine granulocyte count.

In summary, the TyGI was independently and positively correlated with PAD in hospitalized vascular surgery patients aged 50 and older. The findings suggest that the TyGI may serve as a useful indicator for assessing PAD risk, particularly in individuals with metabolic syndrome or other cardiovascular risk factors.

### 3.4 Sensitivity analysis with multiple imputation for missing data

**Supplementary Table 1** demonstrates significant differences between included (N=3,538) and excluded patients (N=2,385) in age, PAD prevalence, ALT, NEUT, and comorbidities (hypertension, diabetes, smoking, and alcohol use). However, key metabolic parameters (BMI, TC, LDL) showed no significant variations. These observed differences likely reflect our exclusion criteria, which eliminated not only patients with missing TyG index data but also those with severe comorbidities or recent use of metabolism-affecting medications.

To address potential bias from missing data, we performed sensitivity analyses using multiple imputation (five imputed datasets). As shown in **Supplementary Table 2**, the association between TyG index and PAD remained statistically significant across all imputed datasets, with consistent effect sizes. In the fully adjusted model (Model 3), the pooled odds ratio (OR) was 1.78 (95% CI: 1.47–2.24), closely aligning with the pre-imputation analysis (OR=1.92, 95% CI: 1.50–2.45). These results demonstrate that our primary findings are robust even after accounting for missing data, reinforcing the reliability of the conclusions.

### 3.5 Subgroup analyses

To assess the robustness of the observed association between the TyGI and PAD, subgroup analyses were conducted by stratifying the population based on several covariates, including age, sex, BMI, smoking, alcohol consumption, hypertension, diabetes, ALT, NEUT, LDL, and TC while adjusting for other relevant covariates not used for stratification (**Table 4**). The results showed consistent positive correlations between the TyGI and PAD across most subgroups. A significant interaction effect between sex and the TyGI in relation to PAD was identified (*P* for interaction < 0.05). In male vascular surgery patients, the TyGI was significantly and positively associated with PAD (OR = 2.27, 95% CI: 1.71–3.02, *P* < 0.001). On the other hand, in female patients, the association between the TyGI and PAD was weaker and not statistically significant (OR = 1.37, 95% CI: 0.92–2.02, *P* = 0.118). These findings suggest a potential sex-specific difference in the relationship between the TyGI and PAD risk.

**Table 4:** Subgroup analyses examining the relationship between TyG index and PAD.

Subgroup	N	OR (95% CI) *P*-value	Interaction P-value
Age, years			
>50, ≤70	2416	2.05 (1.47, 2.86) <0.001	0.427
>70	1122	1.74 (1.28, 2.35) <0.001	
Sex			
Female	1565	1.37 (0.92, 2.02) 0.118	0.024
Male	1973	2.27 (1.71, 3.02) <0.001	
BMI, kg/m^2^			
≤ 24.0	1630	1.84 (1.31, 2.57) <0.001	0.815
>24.0, ≤ 28.0	1437	1.80 (1.13,2.86) 0.014	
>28.0	471	2.47 (0.98, 6.21) 0.054	
Smoking			
No	2418	1.87 (1.39, 2.50) <0.001	0.911
Yes	307	2.12 (1.21, 3.71) 0.008	
Unknown	813	1.95 (1.28, 2.99) 0.002	
Drinking			
No	2534	1.84 (1.39 ,2.43) <0.001	0.445
Yes	191	3.41 (1.32 ,8.79) 0.011	
Unknown	813	1.96 (1.28 ,3.00) 0.002	
Hypertension			
No	2221	1.58 (1.07, 2.35) 0.022	0.213
Yes	1317	2.09 (1.58, 2.77) <0.001	
Diabetes			
No	3174	1.93 (1.44, 2.58) <0.001	0.948
Yes	364	1.90 (1.30, 2.77) <0.001	
TC, mmol/L			
≤5.2	2682	1.93 (1.47, 2.53) <0.001	0.897
>5.2, ≤ 6.2	649	2.09 (1.10, 3.96) 0.024	
>6.2	207	1.69 (0.88, 3.24) 0.114	
LDL, mmol/L			
≤2.6	1613	1.66 (1.21, 2.27) 0.002	0.354
>2.6, ≤ 3.4	1252	2.41 (1.55,3.74) <0.001	
>3.4	673	2.02 (1.21,3.36) 0.007	
NEUT, 10^9/L			
≤6.0	3307	1.98 (1.54, 2.55) <0.001	0.697
>6.0	231	1.74 (0.92, 3.30) 0.088	
ALT, U/L			
≤40.0	3230	1.90 (1.48, 2.44) <0.001	0.674
>40.0	308	2.22 (1.08, 4.60) 0.031	

Patients were stratified based on age, sex, BMI, smoking status, alcohol consumption, hypertension, diabetes, TC, LDL, NEUT, and ALT, and additional covariates not included in the stratification were adjusted for in the analysis.

TyG, triglyceride-glucose; PAD, peripheral artery disease; BMI, body mass index; TC, total cholesterol; LDL, low density lipoprotein; NEUT, medium fine granulocyte count; ALT, alanine aminotransferase.

### 3.6 Spline smoothing plot and threshold analysis

**Figure 2:**
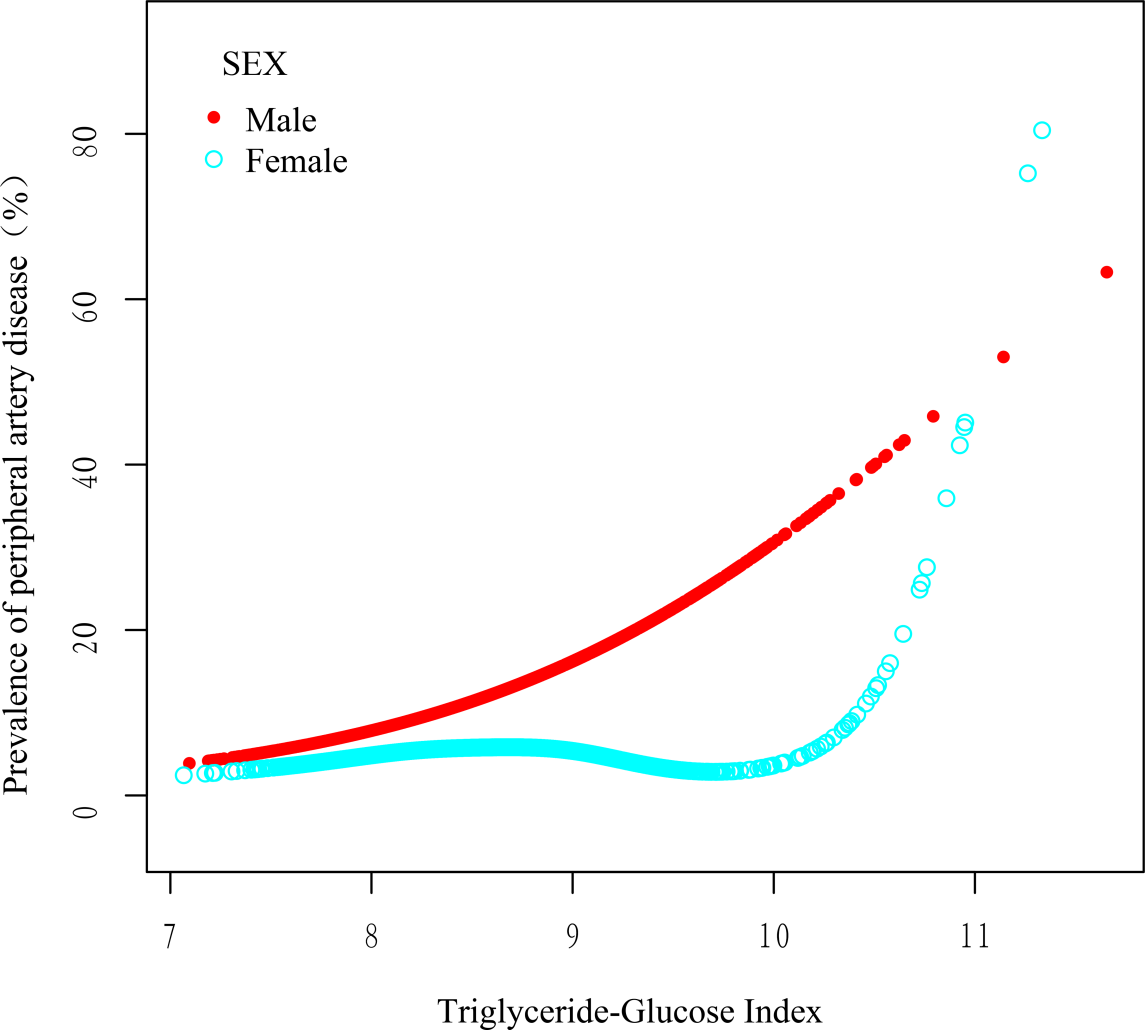
Smoothed adjusted curves illustrating the relationship between the TyGI and PAD. The red line depicts the nonlinear association in male patients, while the blue line shows the nonlinear association in female patients. In female patients, a nonlinear relationship persisted after adjusting for age, sex, BMI, smoking status, alcohol consumption, hypertension, diabetes, LDL, TC, NEUT, and ALT.

To further explore the relationship between the TyGI and PAD, GAM and spline smoothing plots were applied to evaluate potential nonlinear associations. The analysis was stratified by sex (**Table 5**; **Figure 2**). In female vascular surgery patients, the GAM model revealed a nonlinear relationship between the TyGI and PAD after adjusting for confounders. Segmented logistic regression analysis identified an inflection point (K-value) of 9.68. Below this threshold, no significant association between the TyGI and PAD was observed (OR = 0.79, 95% CI: 0.43–1.44, *P* = 0.436). However, above the inflection point, the TyGI showed a positive association with PAD (OR = 11.38, 95% CI: 3.05–42.49, *P* < 0.001), though the wide confidence interval indicates substantial uncertainty in the effect magnitude. In contrast, male vascular surgery patients showed a linear relationship between the TyGI and PAD, with no significant threshold effect detected. This linear association suggests that the risk of PAD increases steadily with higher TyGI levels in male patients.

**Table 5:** Threshold analyses examining the relationship between TyG index and PAD.

Model	Model 3^a^	
	Female	Male
	OR (95%CI) P-value	OR (95%CI) P-value
Model A^b^		
One line slope	1.45 (0.89, 2.36) 0.138	2.11 (1.58, 2.81) <0.001
Model B^c^		
TyG index turning point (K)	9.68	9.61
< K	0.79 (0.43, 1.44) 0.436	1.91 (1.37, 2.64) <0.001
> K	11.38 (3.05, 42.49) <0.001	4.21 (1.40, 12.67) 0.011
Slope 2-Slope 1	14.47 (2.89, 72.59) 0.001	2.21 (0.65, 7.52) 0.206
LRT^d^	<0.001	0.215

a Adjusted for age, sex, BMI, TC, LDL, ALT, NEUT, smoking, drinking, hypertension, diabetes.

b Linear analysis, *P* value<0.05 indicates a linear relationship.

c Nonlinear analysis.

d *P* value<0.05 means Model B is significantly different from Model A, which indicates a nonlinear relationship.

These findings indicate potential sex-based differences in how the TyGI correlates with PAD risk, with a nonlinear threshold effect observed in females and a linear relationship in males. The observed differences may highlight underlying sex-specific pathophysiological mechanisms in PAD development; however, given the wide confidence intervals and imprecision, further validation in larger prospective studies is warranted.

## 4 Discussion

### 4.1 Research summary

This cross-sectional study examined the association between PAD and TyGI in 3,538 vascular surgery inpatients aged 50 years and older. After adjusting for potential confounders, a significant positive correlation between the PAD and TyGI was observed. Importantly, the relationship varied by sex, with a nonlinear correlation in female patients and a linear relationship in male patients. Specifically, in female patients, no significant association was observed when the TyGI was below 9.68, but above this threshold, the association became significant. However, in male patients, the risk of PAD consistently linearly increased with higher TyGI values. These findings suggest sex-specific differences in the pathophysiology of PAD, highlighting the importance of individualized approaches in PAD risk assessment and management.

### 4.2 Relevant studies

The findings of this study are consistent with existing research on the relationship between the TyGI and PAD. Several studies have reported a positive correlation between elevated TyGI levels and an increased risk of PAD, supporting the hypothesis that TyGI may serve as a reliable indicator of IR and atherosclerosis. For instance, a study involving 3,125 participants (mean age 59.9 ± 12.9 years) found that individuals in the highest TyGI quartile had a 1.74-fold increased risk of PAD, even after adjusting for various sociodemographic, lifestyle, and metabolic factors (17). Similarly, an analysis of 12,320 participants without a history of PAD demonstrated that for each standard deviation increase in the TyGI (0.58), the risk of PAD increased by 11.9%. Moreover, participants classified into high and very high stable TyGI trajectory groups showed a greater risk of developing PAD in the future (18). Another study involving 2,027 patients with type 2 diabetes highlighted an interaction effect between the TyGI and systolic blood pressure (SBP), showing that their combination significantly improved PAD prediction (16). These studies emphasize the clinical relevance of the TyGI in predicting PAD risk, particularly in populations with underlying metabolic disorders.

Unlike previous studies, our research focused specifically on vascular surgery inpatients aged over 50 in China, a demographic at high risk for PAD due to age-related metabolic changes. Furthermore, our findings provided novel insights into gender-specific differences, showing a nonlinear relationship in female patients and a consistent linear relationship in male patients. This discrepancy could reflect sex-specific differences in lipid metabolism, hormonal influences, or vascular responses to IR, which warrant further investigation.

### 4.3 Mechanistic insights

#### 4.3.1 Abnormalities in glucose and lipid metabolism

The TyGI, a robust marker of IR, reflects disruptions in glucose and lipid homeostasis (27). IR impairs lipid metabolism, resulting in increased hepatic synthesis of TG within very low-density lipoprotein (VLDL), which subsequently leads to elevated plasma VLDL-TG levels (28). Although this study did not directly measure cholesteryl ester transfer protein (CETP) activity levels, existing evidence suggests that elevated TG concentration modulates CETP activity, promoting the exchange of cholesteryl esters (CE) and TG between lipoproteins (29). This increased transfer rate of CE from HDL to VLDL particles results in a higher abundance of large VLDL particles, which are subsequently metabolized into smaller, denser LDL particles (29). These structurally modified LDL particles demonstrate increased susceptibility to oxidation and reduced affinity for LDL receptors, thereby facilitating their penetration into the arterial intima and ultimately accelerating the formation of atherosclerotic plaques (30–33). Moreover, hormonal changes, particularly in postmenopausal women, exacerbate IR, increasing the likelihood of lipid imbalances and promoting PAD development (34, 35).

Furthermore, differences in fat distribution between men and women, with women tending to have higher visceral fat accumulation, may explain the stronger association between the TyGI and PAD in women. Elevated visceral fat is metabolically active and linked to increased free fatty acids, worsening insulin sensitivity, and lipid metabolism.

#### 4.3.2 Changes in inflammatory responses

Inflammation plays a key role in the progression of atherosclerosis and PAD (36, 37). Elevated inflammatory markers, such as interleukin-6 (IL-6) and tumor necrosis factor-alpha (TNF-α), have been observed in patients with PAD, contributing to endothelial dysfunction and plaque formation (37, 38). The TyGI is positively associated with systemic inflammation, as increased glucose and triglyceride levels stimulate inflammatory cytokine production and oxidative stress (30, 39). Moreover, evidence suggests that the inflammatory response is more significant in women, likely due to higher baseline levels of inflammatory markers and homocysteine (Hcy), which aggravates vascular inflammation (40, 41). This improved inflammatory state may explain why the TyGI shows a nonlinear relationship with PAD in female patients (40). Once a critical threshold of metabolic disturbance is crossed, inflammation and oxidative stress increase rapidly, contributing to the observed nonlinear effect.

#### 4.3.3 Hormonal influences

Estrogen exerts vasculoprotective effects through multiple mechanisms including enhanced vasodilation (42), inhibition of atherosclerotic plaque formation (43), antioxidant and anti-inflammatory activities (44, 45), and improvement of lipid metabolism (43). These protective mechanisms may explain the absence of significant association between TyGI and PAD in women with TyGI levels below the threshold (TyGI <9.68). However, the postmenopausal decline in estrogen levels exacerbates insulin resistance and systemic inflammation (46). Concurrently, the increasing prevalence of metabolic syndrome (characterized by central obesity, dyslipidemia and insulin resistance) among women collectively disrupts metabolic homeostasis (47). When the degree of vascular damage induced by elevated TyGI levels surpasses the compensatory capacity of estrogen’s protective effects, a significant TyGI-PAD association becomes evident in women. These interacting factors likely underlie the observed nonlinear relationship, where PAD risk achieves statistical significance only when exceeding a critical TyGI threshold, reflecting the complex interplay between hormonal protection and metabolic dysregulation in female pathophysiology. It is important to note that, due to the lack of specific data on estrogen levels in women, our discussion regarding estrogen is primarily speculative. Clarification of the precise mechanisms will require future studies that incorporate these parameters for validation.

#### 4.3.4 Endothelial dysfunction

The TyGI is closely related to endothelial dysfunction, a key early event in atherosclerosis and PAD. IR, oxidative stress, and hyperlipidemia impair endothelial cell function, reducing nitric oxide availability and impairing vasodilation. This results in hemodynamic changes, promoting atherosclerotic plaque development and increasing the risk of arterial occlusion (48, 49). PAD patients often show elevated oxidative stress, which damages endothelial cells and promotes lipid peroxidation, further aggravating plaque formation (50, 51). Oxidative stress not only damages vascular endothelial cells but also promotes inflammatory responses and lipid deposition, aggravating the occurrence and progression of PAD (52, 53).

#### 4.3.5 Genes and genetics

Emerging evidence suggests that the TyGI may influence gene expression related to lipid metabolism and inflammation, thereby increasing genetic susceptibility to PAD. Polymorphisms in genes regulating insulin sensitivity, cholesterol metabolism, and inflammatory pathways may modify the association between the TyGI and PAD risk (54). Furthermore, the TyGI correlates positively with hypertension, diabetes, and obesity, which are influenced by genetic predispositions (51, 55). Collectively, these factors create a polygenic risk environment where individuals with high TyGI levels are more likely to develop PAD. Future studies should focus on elucidating the genetic and epigenetic mechanisms underlying the TyG index’s role in PAD, particularly in different sex groups.

### 4.4 Clinical applications

The study’s findings have significant clinical relevance. The observed positive correlation between the TyGI and PAD, particularly the threshold effect and nonlinear relationship in females, highlights the need for more personalized risk assessment strategies. Specifically, when the TyGI exceeds 9.68 in females, the risk of PAD increases. This suggests that there may be protective mechanisms in female patients with a lower TyGI that mitigate the adverse effects on peripheral vascular health. Once this threshold is surpassed, however, the vascular risks rise.

Routine monitoring of the TyGI can offer clinicians a simple and non-invasive method to assess PAD risk in hospitalized vascular surgery patients. Moreover, timely intervention in patients with a TyGI above the critical threshold could potentially reduce PAD incidence, enhance peripheral vascular health, and improve overall quality of life. Given the association of the TyGI with other cardiovascular diseases, its clinical use could extend beyond PAD to support comprehensive cardiovascular risk management.

### 4.5 Study advantages and limitations

This study has several strengths. The study employed three distinct models—unadjusted, minimally adjusted, and fully adjusted—to comprehensively explore the relationship between the TyGI and PAD. This approach minimized the effect of potential confounders and provided robust evidence for the observed associations. Moreover, unlike the ABI, which requires specialized equipment and trained personnel, the TyGI can be derived from routine blood tests, making it more accessible and feasible for large-scale clinical use. This enhances its potential as a screening tool for PAD, especially in high-risk hospitalized populations. However, the study has limitations. First, since the study is cross-sectional, it cannot establish causality. Therefore, while a strong association between TyGI and PAD was observed, it remains unclear whether elevated TyGI levels directly contribute to PAD progression or are merely indicative of an underlying condition. Longitudinal studies are needed to establish a temporal relationship. Second, patients taking medications that influence glucose and lipid metabolism were excluded from the study. While this approach helped isolate the natural relationship between the TyGI and PAD, it limits the generalizability of the findings to populations on such medications, which are common in clinical settings. Third, although 3,538 patients were included, the sample size may still be insufficient to detect subtle subgroup differences or interactions. Additionally, 2,176 patients were excluded due to missing TYGI data. Although multiple imputation analyses supported the robustness of our findings, we cannot entirely rule out the potential influence of this missing data on the results. Moreover, since the study was conducted in a single hospital, the results may not apply to other ethnic groups or healthcare settings. Multicenter studies with more diverse populations are necessary to confirm these findings and expand their applicability. Fourth, This study hypothesizes hormonal, inflammatory, and lipid metabolic mechanisms underlying the association between TyGI and PAD. However, due to data limitations, key biological markers such as estrogen levels, C-reactive protein (CRP), and CETP activity were not measured, which restricts the ability to directly investigate these mechanisms or provide robust supporting evidence. Although NEUT was used as a marker of inflammation, it has limited specificity and sensitivity for inflammatory processes. Future studies should incorporate systematic measurement of these biomarkers and consider mediation analyses to better elucidate the pathways involved. Fifth, PAD is influenced by numerous genetic and environmental factors. Although key variables were adjusted for, unmeasured factors such as dietary patterns, physical activity, and genetic predisposition could have influenced the results. Including broader biochemical markers and genetic data in future studies could enhance understanding of TyGI’s role in PAD. Finally, while a threshold effect was identified in female patients, the exact biological mechanism underlying this effect remains unclear. Further mechanistic studies are needed to explore why this nonlinear relationship exists in women but not in men.

## 5 Conclusion

In conclusion, this study highlights the TyGI as a significant predictor for PAD risk, with a distinct influence of sex on the relationship. A nonlinear correlation between the TyGI and PAD was observed in female patients, with a critical threshold effect at a TyGI of 9.68, while in male patients, a linear positive correlation was consistently observed. These findings highlight the need for sex-specific risk assessment strategies in clinical practice to improve early detection and intervention for PAD. Despite the robust statistical models employed, this study had several limitations, including the relatively small sample size, cross-sectional design, unmeasured potential confounders, and a focus on a specific population (hospitalized vascular surgery patients). Moreover, the absence of mechanistic studies limits our understanding of the biological pathways underlying the observed sex differences. Therefore, further research—particularly longitudinal and mechanistic studies—is necessary to validate these findings, explore potential underlying mechanisms, and develop more targeted strategies for PAD management. By integrating the TyGI into routine clinical evaluations, especially in high-risk populations, clinicians can improve PAD screening and potentially mitigate the burden of this disease through earlier and more personalized interventions.

## Data Availability

The raw data supporting the conclusions of this article will be made available by the authors, without undue reservation.

## References

[1] Grenon SM Vittinghoff E Owens CD Conte MS Whooley M Cohen BE. Peripheral artery disease and risk of cardiovascular events in patients with coronary artery disease: Insights from the Heart and Soul Study. Vasc Med. (2013) 18:176–84. doi: 10.1177/1358863X13493825, PMID: 23835937 PMC4207208

[2] Pérez Mejia EL Faxas SM Taveras NT Talpur AS Jitesh K Khalid M. Peripheral artery disease as a risk factor for myocardial infarction. Cureus. (2021) 13(6):e15655. doi: 10.7759/cureus.15655, PMID: 34277248 PMC8280959

[3] Fowkes FGR Aboyans V Fowkes FJI McDermott MM Sampson UKA Criqui MH. Peripheral artery disease: epidemiology and global perspectives. Nat Rev Cardiol. (2017) 14:156–70. doi: 10.1038/nrcardio.2016.179, PMID: 27853158

[4] Subramaniam T Nang EEK Lim SC Wu Y Khoo CM Lee J. Distribution of ankle—brachial index and the risk factors of peripheral artery disease in a multi-ethnic Asian population. Vasc Med. (2011) 16:87–95. doi: 10.1177/1358863X11400781, PMID: 21447605

[5] Yi C Jie C Junyi G Fengju L Qing Z. Association between lipoprotein(a) and peripheral arterial disease in coronary artery bypass grafting patients. Clin Cardiol. (2023) 46(5):512–20. doi: 10.1002/clc.24003, PMID: 36896666 PMC10189068

[6] Tikkanen E Jägerroos V Holmes MV Sattar N Ala-Korpela M Jousilahti P. Metabolic biomarker discovery for risk of peripheral artery disease compared with coronary artery disease: lipoprotein and metabolite profiling of 31–657 individuals from 5 prospective cohorts. J Am Heart Assoc. (2021) 10:e021995. doi: 10.1161/JAHA.121.021995, PMID: 34845932 PMC9075369

[7] Miao C Fang X Chen Y Zhang Q. Effect of vitamin D supplementation on polycystic ovary syndrome: a meta−analysis. Exp Ther Med. (2020) 19(4):2641–9. doi: 10.3892/etm.2020.8525, PMID: 32256745 PMC7086222

[8] Alzahrani HA Wang D Bakhotmah BA Hu FB. Risk factors for peripheral artery disease among patients with diabetes in Saudi Arabia. Vasc Med. (2014) 19:103–11. doi: 10.1177/1358863X14526948, PMID: 24621989

[9] Rein P Saely CH Silbernagel G Vonbank A Mathies R Drexel H. Systemic inflammation is higher in peripheral artery disease than in stable coronary artery disease. Atherosclerosis. (2015) 239:299–303. doi: 10.1016/j.atherosclerosis.2015.01.021, PMID: 25682027

[10] Hussein AA Uno K Wolski K Kapadia S Schoenhagen P Tuzcu EM. Peripheral arterial disease and progression of coronary atherosclerosis. J Am Coll Cardiol. (2011) 57(10):1220–5:. doi: 10.1016/j.jacc.2010.10.034, PMID: 21371639

[11] Eraso LH Fukaya E Mohler ER Xie D Sha D Berger JS. Peripheral arterial disease, prevalence and cumulative risk factor profile analysis. Eur J Prev Cardiol. (2014) 21:704–11. doi: 10.1177/2047487312452968, PMID: 22739687 PMC4436703

[12] Guerrero-Romero F Simental-Mendía LE González-Ortiz M Martínez-Abundis E Ramos-Zavala MG Hernández-González SO. The product of triglycerides and glucose, a simple measure of insulin sensitivity. Comparison with the euglycemic-hyperinsulinemic clamp. J Clin Endocrinol Metab. (2010) 95:3347–51. doi: 10.1210/jc.2010-0288, PMID: 20484475

[13] Khan SH Sobia F Niazi NK Manzoor SM Fazal N Ahmad F. Metabolic clustering of risk factors: evaluation of Triglyceride-glucose index (TyG index) for evaluation of insulin resistance. Diabetol Metab Syndr. (2018) 10:74. doi: 10.1186/s13098-018-0376-8, PMID: 30323862 PMC6173832

[14] Hou Q Qi Q Han Q Yu J Wu J Yang H. Association of the triglyceride-glucose index with early-onset atherosclerotic cardiovascular disease events and all-cause mortality: a prospective cohort study. Cardiovasc Diabetol. (2024) 23:149. doi: 10.1186/s12933-024-02249-4, PMID: 38685099 PMC11059708

[15] Chen L Ding X-H Fan K-J Gao M-X Yu W-Y Liu H-L. Association between triglyceride-glucose index and 2-year adverse cardiovascular and cerebrovascular events in patients with type 2 diabetes mellitus who underwent off-pump coronary artery bypass grafting. Diabetes Metab Syndr Obes Targets Ther. (2022) 15:439–50. doi: 10.2147/DMSO.S343374, PMID: 35210794 PMC8858766

[16] Gong C Chen C Zhao Y Wang Y Li K Lv X. Interaction and combined effect of triglyceride-glucose index and hypertension on type 2 diabetes individuals’ peripheral arterial disease risk. Acta Diabetol. (2024) 62(5):717–29. doi: 10.1007/s00592-024-02391-1, PMID: 39460758

[17] Liu Y Chang L Wu M Xu B Kang L. Triglyceride glucose index was associated with the risk of peripheral artery disease. Angiology. (2022) 73:655–9. doi: 10.1177/00033197211070644, PMID: 35077252

[18] Gao J-W Hao Q-Y Gao M Zhang K Li X-Z Wang J-F. Triglyceride-glucose index in the development of peripheral artery disease: findings from the Atherosclerosis Risk in Communities (ARIC) Study. Cardiovasc Diabetol. (2021) 20:126. doi: 10.1186/s12933-021-01319-1, PMID: 34167539 PMC8223290

[19] Hamur H Onk OA Vuruskan E Duman H Bakirci EM Kucuksu Z. Determinants of chronic total occlusion in patients with peripheral arterial occlusive disease. Angiology. (2017) 68(2):151–8. doi: 10.1177/0003319716641827, PMID: 27059289

[20] Lee C-C Wu C-J Chou L-H Shen S-M Chiang S-F Jen P-C. Peripheral artery disease in peritoneal dialysis and hemodialysis patients: single-center retrospective study in Taiwan. BMC Nephrol. (2012) 13:100. doi: 10.1186/1471-2369-13-100, PMID: 22943313 PMC3447712

[21] Aboyans V Ricco JB Bartelink MEL Björck M Brodmann M Cohnert T. 2017 ESC Guidelines on the diagnosis and treatment of peripheral arterial diseases, in collaboration with the European Society for Vascular Surgery (ESVS): document covering atherosclerotic disease of extracranial carotid and vertebral, mesenteric, renal, upper and lower extremity arteries. Eur Heart J. (2018) 39(9):763–816. doi: 10.1093/eurheartj/ehx095, PMID: 28886620

[22] Gerhard-Herman MD Gornik HL Barrett C Barshes NR Corriere MA Drachman DE. 2016 AHA/ACC guideline on the management of patients with lower extremity peripheral artery disease: executive summary: A report of the american college of cardiology/american heart association task force on clinical practice guidelines. Circulation. (2017) 135(12):e686–725. doi: 10.1161/CIR.0000000000000470, PMID: 27840332 PMC5479414

[23] Song Y Zhao Y Shu Y Zhang L Cheng W Wang L. Combination model of neutrophil to high-density lipoprotein ratio and system inflammation response index is more valuable for predicting peripheral arterial disease in type 2 diabetic patients: A cross-sectional study. Front Endocrinol. (2023) 14:1100453. doi: 10.3389/fendo.2023.1100453, PMID: 36875480 PMC9978802

[24] Khlat M Van Cleemput O Bricard D Legleye S. Use of tobacco, alcohol and cannabis in late adolescence: roles of family living arrangement and socioeconomic group. BMC Public Health. (2020) 20:1356. doi: 10.1186/s12889-020-09476-w, PMID: 32887597 PMC7650265

[25] He L Yan Y Wang Y Sun Y La Y Liu J. Identifying excessive intake of oil and salt to prevent and control hypertension: A latent class analysis. Front Cardiovasc Med. (2022) 9:782639. doi: 10.3389/fcvm.2022.782639, PMID: 35463793 PMC9019702

[26] Xu J Guo S Xu M Li C Gong Y Lu K. The association between the triglyceride-glucose index and bone turnover markers in osteoporotic fractures patients aged 50 and above who are hospitalized for surgical intervention: a retrospective cross-sectional study. Front Endocrinol. (2024) 15:1418271. doi: 10.3389/fendo.2024.1418271, PMID: 39359411 PMC11445018

[27] Sun Q Liu J Meng R Zhang N Yao J Yang F. Association of the triglyceride-glucose index with subclinical left ventricular dysfunction in type 2 diabetes mellitus patients: A retrospective cross-sectional study. J Diabetes Investig. (2023) 14:953–60. doi: 10.1111/jdi.14026, PMID: 37151188 PMC10360383

[28] Bjornstad P Eckel RH. Pathogenesis of lipid disorders in insulin resistance: a brief review. Curr Diabetes Rep. (2018) 18:127. doi: 10.1007/s11892-018-1101-6, PMID: 30328521 PMC6428207

[29] Ito F Ito T. High-density lipoprotein (HDL) triglyceride and oxidized HDL: new lipid biomarkers of lipoprotein-related atherosclerotic cardiovascular disease. Antioxidants. (2020) 9:362. doi: 10.3390/antiox9050362, PMID: 32357465 PMC7278571

[30] Leiherer A Mündlein A Brandtner EM Säly CH Ramadani H Vonbank A. Lipid profiles of patients with manifest coronary versus peripheral atherosclerosis – Is there a difference? J Intern Med. (2021) 290:1249–63. doi: 10.1111/joim.13368, PMID: 34337800

[31] Klop B Elte J Cabezas M. Dyslipidemia in obesity: mechanisms and potential targets. Nutrients. (2013) 5:1218–40. doi: 10.3390/nu5041218, PMID: 23584084 PMC3705344

[32] Austin MA. Genetic epidemiology of dyslipidaemia and atherosclerosis. Ann Med. (1996) 28:459–63. doi: 10.3109/07853899608999108, PMID: 8949979

[33] Kondo A Muranaka Y Ohta I Notsu K Manabe M Kotani K. Relationship between triglyceride concentrations and LDL size evaluated by malondialdehyde-modified LDL. Clin Chem. (2001) 47:893–900. doi: 10.1093/clinchem/47.5.893, PMID: 11325894

[34] Lee EY Yang HK Lee J Kang B Yang Y Lee S-H. Triglyceride glucose index, a marker of insulin resistance, is associated with coronary artery stenosis in asymptomatic subjects with type 2 diabetes. Lipids Health Dis. (2016) 15:155. doi: 10.1186/s12944-016-0324-2, PMID: 27633375 PMC5024477

[35] Cui C Liu L Zhang T Fang L Mo Z Qi Y. Triglyceride-glucose index, renal function and cardiovascular disease: a national cohort study. Cardiovasc Diabetol. (2023) 22:325. doi: 10.1186/s12933-023-02055-4, PMID: 38017519 PMC10685637

[36] Gardner AW Parker DE Montgomery PS Sosnowska D Casanegra AI Ungvari Z. Greater endothelial apoptosis and oxidative stress in patients with peripheral artery disease. Int J Vasc Med. (2014) 2014:1–8. doi: 10.1155/2014/160534, PMID: 24963409 PMC4054861

[37] Signorelli SS Anzaldi M Libra M Navolanic PM Malaponte G Mangano K. Plasma levels of inflammatory biomarkers in peripheral arterial disease: results of a cohort study. Angiology. (2016) 67:870–4. doi: 10.1177/0003319716633339, PMID: 26888895

[38] Fort-Gallifa I Hernández-Aguilera A García-Heredia A Cabré N Luciano-Mateo F Simó J. Galectin-3 in peripheral artery disease. Relationships with markers of oxidative stress and inflammation. Int J Mol Sci. (2017) 18:973. doi: 10.3390/ijms18050973, PMID: 28471381 PMC5454886

[39] Li C Kitzerow O Nie F Dai J Liu X Carlson MA. Bioengineering strategies for the treatment of peripheral arterial disease. Bioact Mater. (2021) 6:684–96. doi: 10.1016/j.bioactmat.2020.09.007, PMID: 33005831 PMC7511653

[40] Zhao Q Zhang T-Y Cheng Y-J Ma Y Xu Y-K Yang J-Q. Impacts of triglyceride-glucose index on prognosis of patients with type 2 diabetes mellitus and non-ST-segment elevation acute coronary syndrome: results from an observational cohort study in China. Cardiovasc Diabetol. (2020) 19:108. doi: 10.1186/s12933-020-01086-5, PMID: 32641127 PMC7341665

[41] Zhao J Fan H Wang T Yu B Mao S Wang X. TyG index is positively associated with risk of CHD and coronary atherosclerosis severity among NAFLD patients. Cardiovasc Diabetol. (2022) 21:123. doi: 10.1186/s12933-022-01548-y, PMID: 35778734 PMC9250269

[42] Dubey RK Oparil S Imthurn B Jackson EK. Sex hormones and hypertension. Cardiovasc Res. (2002) 53(3):688–708. doi: 10.1016/S0008-6363(01)00527-2 11861040

[43] Bourassa PA Milos PM Gaynor BJ Breslow JL Aiello RJ. Estrogen reduces atherosclerotic lesion development in apolipoprotein E-deficient mice. Proc Natl Acad Sci. (1996) 93:10022–7. doi: 10.1073/pnas.93.19.10022, PMID: 8816744 PMC38329

[44] Lan XF Zhang XJ Lin YN Wang Q Xu HJ Zhou LN. Estradiol regulates txnip and prevents intermittent hypoxia-induced vascular injury. Sci Rep. (2017) 7:10318. doi: 10.1038/s41598-017-10442-7, PMID: 28871193 PMC5583380

[45] Chou S-H Lee Y-C Huang C-F Wang Y-R Yu H-P Lau Y-T. Gender-specific effects of caloric restriction on the balance of vascular nitric oxide and superoxide radical. Cardiovasc Res. (2010) 87:751–9. doi: 10.1093/cvr/cvq095, PMID: 20348138

[46] Lee SB Kim MK Kang S Park K Kim JH Baik SJ. Triglyceride glucose index is superior to the homeostasis model assessment of insulin resistance for predicting nonalcoholic fatty liver disease in Korean adults. Endocrinol Metab. (2019) 34:179. doi: 10.3803/EnM.2019.34.2.179, PMID: 31257745 PMC6599902

[47] Li R Li W Lun Z Zhang H Sun Z Kanu JS. Prevalence of metabolic syndrome in mainland China: a meta-analysis of published studies. BMC Public Health. (2016) 16:296. doi: 10.1186/s12889-016-2870-y, PMID: 27039079 PMC4818385

[48] Hart CR Layec G Trinity JD Kwon OS Zhao J Reese VR. Increased skeletal muscle mitochondrial free radical production in peripheral arterial disease despite preserved mitochondrial respiratory capacity. Exp Physiol. (2018) 103:838–50. doi: 10.1113/EP086905, PMID: 29604234 PMC7640985

[49] Gardner AW Parker DE Montgomery PS Sosnowska D Casanegra AI Esponda OL. Impaired vascular endothelial growth factor A and inflammation in patients with peripheral artery disease. Angiology. (2014) 65:683–90. doi: 10.1177/0003319713501376, PMID: 24006146 PMC4043949

[50] Ismaeel A Papoutsi E Miserlis D Lavado R Haynatzki G Casale GP. The nitric oxide system in peripheral artery disease: connection with oxidative stress and biopterins. Antioxidants. (2020) 9:590. doi: 10.3390/antiox9070590, PMID: 32640613 PMC7402092

[51] Keramat S Sharebiani H Patel M Fazeli B Stanek A. The potential role of antioxidants in the treatment of peripheral arterial disease: A systematic review. Antioxidants. (2022) 11:2126. doi: 10.3390/antiox11112126, PMID: 36358498 PMC9686635

[52] Drew RC Muller MD Blaha CA Mast JL Heffernan MJ Estep LE. Renal vasoconstriction is augmented during exercise in patients with peripheral arterial disease. Physiol Rep. (2013) 1:e00154. doi: 10.1002/phy2.154, PMID: 24400156 PMC3871469

[53] Koutakis P Ismaeel A Farmer P Purcell S Smith RS Eidson JL. Oxidative stress and antioxidant treatment in patients with peripheral artery disease. Physiol Rep. (2018) 6:e13650. doi: 10.14814/phy2.13650, PMID: 29611350 PMC5880878

[54] Koutakis P Hernandez H Miserlis D Thompson JR Papoutsi E Mietus CJ. Oxidative damage in the gastrocnemius predicts long-term survival in patients with peripheral artery disease. NPJ Aging. (2024) 10:21. doi: 10.1038/s41514-024-00147-3, PMID: 38580664 PMC10997596

[55] Ismaeel A Brumberg RS Kirk JS Papoutsi E Farmer PJ Bohannon WT. Oxidative stress and arterial dysfunction in peripheral artery disease. Antioxidants. (2018) 7:145. doi: 10.3390/antiox7100145, PMID: 30347720 PMC6210426

