# Supplementary material for: Gender differences in the association between the triglyceride-glucose index and peripheral artery disease in vascular surgery inpatients aged 50 and above: a retrospective cross-sectional study
Source: Front Endocrinol (Lausanne). 2025 Aug 20;16:1578025. doi: 10.3389/fendo.2025.1578025 (PMC12404938; doi:10.3389/fendo.2025.1578025)
Supplement: Supplementary file 1 [file Table1.docx]

Supplementary Table 1. Patient characteristics based on inclusion and exclusion for the study.

|  | Mean±SD / N (%) | |  |  |
| --- | --- | --- | --- | --- |
|  | Excluded patients | Include patients | P-value | P-value* |
| N | 2385 | 3538 |  |  |
| TyG index | - | 8.62±0.60 | - | - |
| Age, years | 68.48±9.98 | 66.29±9.13 | <0.001 | <0.001 |
| BMI, kg/m^2^ | 24.25±3.61 | 24.32±3.31 | 0.502 | 0.360 |
| TC, mmol/L | 4.26±1.01 | 4.55±1.04 | 0.277 | 0.433 |
| TG, mmol/L | - | 1.43±0.99 | - | - |
| LDL, mmol/L | 2.54±0.88 | 2.72±0.82 | 0.380 | 0.501 |
| ALT, U/L | 26.80±31.54 | 23.35±17.42 | <0.001 | <0.001 |
| NEUT, 10^9/L | 5.13±3.26 | 3.75±1.71 | <0.001 | <0.001 |
| FBG, mmol/L | - | 5.82±1.63 | - | - |
| N (%) | | | | |
| Sex, N (%) |  |  | 0.110 | - |
| Female | 1005（42.14%） | 1565（44.23%） |  |  |
| Male | 1380（57.86%） | 1973（55.77%） |  |  |
| Smoking, N (%) |  |  | <0.001 | - |
| No | 1883（78.95%） | 2418（68.34%） |  |  |
| Yes | 254（10.65%） | 307（8.68%） |  |  |
| Unknown | 248（10.40%） | 813（22.98%） |  |  |
| Drinking, N (%) |  |  | <0.001 | - |
| No | 1973（82.73%） | 2534（71.62%） |  |  |
| Yes | 165（6.92%） | 191（5.40%） |  |  |
| Unknown | 247（10.36%） | 813（22.98%） |  |  |
| Hypertension, N (%) |  |  | <0.001 | - |
| No | 1232（51.66%） | 2221（62.78%） |  |  |
| Yes | 1153（48.34%） | 1317（37.22%） |  |  |
| Diabetes, N (%) |  |  | <0.001 | - |
| No | 2025（84.91%） | 3174（89.71%） |  |  |
| Yes | 360（15.09%） | 364（10.29%） |  |  |
| PAD, % |  |  | 0.019 | - |
| No | 2129(89.27%) | 3223(91.10%) |  |  |
| Yes | 256(10.73%) | 315(8.90%) |  |  |

TyG, triglyceride-glucose index; SD, standard deviation; Q1, first quartile; Q2, second quartile; Q3, third quartile; Q4, fourth quartile; BMI, body mass index; TC, total cholesterol; TG, triglyceride; LDL, low density lipoprotein; ALT, alanine aminotransferase; NEUT, medium fine granulocyte count; FBG, fasting blood glucose; PAD, peripheral artery disease.

P-value*: Kruskal Wallis Rank Test for continuous variables, Fisher Exact for categorical variables with Expects<10.
