# Supplementary material for: Gender differences in the association between the triglyceride-glucose index and peripheral artery disease in vascular surgery inpatients aged 50 and above: a retrospective cross-sectional study
Source: Front Endocrinol (Lausanne). 2025 Aug 20;16:1578025. doi: 10.3389/fendo.2025.1578025 (PMC12404938; doi:10.3389/fendo.2025.1578025)
Supplement: Supplementary file 2 [file Table2.docx]

Supplementary Table 2. Regression analysis results from multiple imputation dataset.

| TyG index | Cases/n | Model 1^a^ | Model 2^b^ | Model 3^c^ |
| --- | --- | --- | --- | --- |
|  |  | OR (95%CI)  P-value | OR (95%CI)  P-value | OR (95%CI)  P-value |
| Multiple Imputation Dataset 1 | 571/5923 | 1.21 (1.05, 1.41) 0.010 | 1.49 (1.27, 1.74) <0.001 | 1.81 (1.47, 2.24) <0.001 |
| Multiple Imputation Dataset 2 | 571/5923 | 1.17 (1.01, 1.35) 0.042 | 1.44 (1.23, 1.68) <0.001 | 1.72 (1.40, 2.13) <0.001 |
| Multiple Imputation Dataset 3 | 571/5923 | 1.22 (1.05, 1.42) 0.009 | 1.52 (1.29, 1.78) <0.001 | 1.72 (1.39, 2.12) <0.001 |
| Multiple Imputation Dataset 4 | 571/5923 | 1.21 (1.05, 1.40) 0.109 | 1.50 (1.29, 1.75) <0.001 | 1.80 (1.46, 2.23) <0.001 |
| Multiple Imputation Dataset 5 | 571/5923 | 1.24 (1.07, 1.44) 0.005 | 1.54 (1.32, 1.81) <0.001 | 1.85 (1.50, 2.28) <0.001 |
| mean value of multiple input datasets |  | 1.21 | 1.50 | 1.78 |
| Pre-Imputation Dataset | 315/3538 | 1.25 (1.03, 1.51) 0.020 | 1.75 (1.44, 2.13) <0.001 | 1.92 (1.50, 2.45) <0.001 |

^a^No adjustment.

^b^Adjusted for age, sex.

^c^Adjusted for age, sex, BMI, TC, LDL, ALT, NEUT, smoking, drinking, hypertension, diabetes.

TyG, triglyceride-glucose; PAD, peripheral artery disease; BMI, body mass index; TC, total cholesterol; LDL, low density lipoprotein; ALT, alanine aminotransferase; NEUT, medium fine granulocyte count.
